# Social Media Exposure and Other Correlates of Increased e-Cigarette Use Among Adolescents During Remote Schooling: Cross-Sectional Study

**DOI:** 10.2196/49779

**Published:** 2024-10-21

**Authors:** Vira Pravosud, Pamela M Ling, Bonnie Halpern-Felsher, Valerie Gribben

**Affiliations:** 1 Center for Tobacco Control Research and Education, University of California San Francisco, San Francisco, CA, United States; 2 Center for Data to Discovery and Delivery Innovation, San Francisco VA Health Care System, San Francisco, CA, United States; 3 Northern California Institute for Research and Education, San Francisco, CA, United States; 4 School of Medicine, Department of Medicine, Division of General Internal Medicine, University of California San Francisco, San Francisco, CA, United States; 5 REACH Lab, Division of Adolescent Medicine, Department of Pediatrics, Stanford University, Palo Alto, CA, United States; 6 Department of Pediatrics, University of California San Francisco, San Francisco, CA, United States

**Keywords:** adolescents, social media use, e-cigarette use, mental health, COVID-19 shelter-in-place orders, remote schooling, smoking, vape, e-cigarette implications, COVID-19, anxiety, depression

## Abstract

**Background:**

Little is known about the role of exposure to e-cigarette–related digital content, behavioral and mental health factors, and social environment on the change in adolescent e-cigarette use during COVID-19 shelter-in-place orders and remote schooling.

**Objective:**

The aim of the study was to examine changes in adolescent e-cigarette use during shelter-in-place and remote schooling in association with exposure to e-cigarette–related digital content and other correlates: stronger e-cigarette dependence, feeling lonely, inability to socialize, e-cigarette use to cope with shelter-in-place, and the number of family members aware of participants’ e-cigarette use.

**Methods:**

A cross-sectional survey conducted between August 2020 and March 2021 included 85 California adolescents (mean age 16.7, SD 1.2 years; 39/85, 46% identified as female and 37/85, 44% as Hispanic) who reported e-cigarette use in the past 30 days. Multivariable penalized logistic regressions determined associations adjusted for age, race and ethnicity, and mother’s education. The outcome of increased e-cigarette use was defined as more frequent use of e-cigarettes of the same or stronger nicotine or tetrahydrocannabinol concentration.

**Results:**

Almost all respondents (83/85, 98%) reported using social media more since shelter-in-place, and 74% (63/85) reported seeing e-cigarette digital content. More than half (46/85, 54%) reported increased e-cigarette use during shelter-in-place. Most individuals who increased use were exposed to e-cigarette digital content (38/46, 83%) compared to those who did not increase e-cigarette use (25/39, 64%), but the association was nonsignificant after adjusting for demographics (adjusted odds ratio [AOR] 2.34, 95% CI 0.71‐8.46). Respondents who felt lonely (AOR 3.33, 95% CI 1.27‐9.42), used e-cigarettes to cope with shelter-in-place (AOR 4.06, 95% CI 1.39‐13.41), or had ≥2 family members aware of participants’ e-cigarette use (AOR 6.42, 95% CI 1.29‐39.49) were more likely to report increased e-cigarette use.

**Conclusions:**

Almost all participants reported using social media more during shelter-in-place, with many respondents reporting increased e-cigarette use, and significant associations with loneliness and use to cope with shelter-in-place. Future interventions should consider leveraging digital platforms for e-cigarette use prevention and cessation and address the mental health consequences of the COVID-19 pandemic.

## Introduction

### Background

In 2021, 3.3% of middle and 14.1% of high school students in the United States reported e-cigarette use in the past 30 days [1]. Feelings of anxiety, depression, or stress (43.4%) and the use of e-cigarettes by friends (28.3%) are commonly cited reasons for adolescent e-cigarette use [2]. Harmful effects on the developing brain and lungs [3] and a higher risk of addiction to nicotine and other drugs [4,5] are some of the adverse health outcomes associated with youth e-cigarette use [6,7]. In addition to nicotine e-cigarettes, the 2021 Monitoring the Future national survey [8] revealed past 30-day use of tetrahydrocannabinol (THC) cannabis e-cigarettes among 4.7% of 8th graders, 12.4% of 10th graders, and 18.3% of 12th graders; and studies in adolescents have shown concurrent use or couse of both nicotine and cannabis [9-12]. Such use of nicotine and THC vaporizers is worrisome due to the hazardous health effects of not only nicotine [13-15] but also THC use [16-18] and potentially elevated health risks associated with couse of both products [19,20].

e-Cigarette use among US adolescents remains a concern [1,21] despite declines in prevalence from 2020 to 2021 [22,23]. The decrease in adolescent use might be related, among other factors [24-26], to the increased public awareness about COVID-19 [23,27,28] and the impact of shelter-in-place orders in the early stage of the pandemic [28,29] (hereafter referred to as “shelter-in-place”). At the same time, both quantitative [28] and qualitative [30] studies reported *increased* youth e-cigarette use due to boredom, stress, or as a distraction during shelter-in-place [23].

A meteoric rise of social media use and prolonged screen time accompanied the COVID-19 pandemic [31]. Frequency of social media use is positively correlated with exposure to e-cigarette–related digital content that, in its turn, is associated with positive attitudes toward e-cigarette use [32]. Observational [33-39] and experimental [40,41] studies have shown that social media use and exposure to social media content (eg, advertisements or posts) are associated with increased willingness and intention to use e-cigarettes [40], increased curiosity [41] and odds of experimental [41,42] and subsequent [38] e-cigarette use among e-cigarette naïve adolescents, greater perceived norms [40] and benefits of e-cigarettes [42], lower perceived danger [39,40], and more positive attitudes toward e-cigarettes among youths and adolescents [40]. Increased prevalence of adolescent cannabis use has also been associated with exposure to social media cannabis marketing [43].

### Goal of This Study

The need to reduce exposure to e-cigarette–related digital content on social media to prevent tobacco initiation has been raised [44,45], but little is known about the effect of exposure to e-cigarette–related digital content on the change in e-cigarette use among adolescents using tobacco.

To our knowledge, this cross-sectional study is the first to assess the association between exposure to e-cigarette–related digital content on social media and increased e-cigarette use during the unique time frame of shelter-in-place and remote schooling among California adolescents currently using e-cigarettes. Prior research has shown that e-cigarette use is associated with secondhand smoke exposure among family and friends and a pro–e-cigarette social environment [46-48] as well as with mental health and psychological distress [49-52]. Thus, we also aimed to determine potential correlates of increased e-cigarette use during shelter-in-place, including the level of e-cigarette dependence, feeling lonely, inability to socialize during shelter-in-place, e-cigarette use to cope with shelter-in-place, and awareness of family members of participants’ e-cigarette use.

## Methods

### Study Design and Recruitment

This was a cross-sectional study with a convenience sample of adolescents (N=85) who provided their responses to a web-based survey between August 2020 and March 2021. The eligibility criteria included being a middle or high school student in California before California started shelter-in-place on March 19, 2020 [53] and who reported current (past 30-day) use of any e-cigarette products containing nicotine (eg, disposable or pod-based) or THC (eg, marijuana vaporizers and “weed pens”).

SIS International Research recruited adolescent participants by reaching out to their research panels and by posting the study screener on the web. To qualify for the study, adolescents had to be ages 13‐18 years, attending middle or high school in California, and using e-cigarettes at the start of the COVID-19 pandemic. SIS verified adolescents’ age and demographics by reviewing supporting documentation. The research team pilot-tested the survey questionnaires, which were administered anonymously on the Qualtrics platform (Qualtrics, Provo, UT) and designed to take approximately 20 minutes to complete.

### Ethical Considerations

The study was approved by the University of California, San Francisco Institutional Review Board (20‐31136). In the first few months of the study, adolescents provided assent, and a parent or legal guardian provided informed consent, but subsequently, adolescents were later allowed to consent for themselves, consistent with California law, which allows adolescents to consent to medical treatment for substance abuse. Participants received a US $20 gift card incentive. Each participant was assigned a unique survey identification number, and SIS kept their identities confidential. To validate entries of deidentified data, the research team manually checked each completed survey based on geolocation, duration of survey completion, quality of responses to open questions, as well as demographic data (age, gender, and race). Among 126 entries received, 97 were valid responses. We then eliminated 12 duplicate observations for 9 participants, retaining only the response with a longer survey duration time. The final analytic sample included 85 participants with valid responses, who completed the survey between August 12, 2020, and March 4, 2021, during remote schooling; shelter-in-place orders in California were lifted effective June 15, 2021 [54].

### Measures: Outcome and Exposure of Interest

The survey items assessing changes in the frequency of use and concentration of e-cigarettes had the potential to directly demonstrate the impact of shelter-in-place by asking: “Overall, have you changed HOW MUCH you vape since the Shelter-in-Place rules?” and “Overall, has the STRENGTH of your vape changed since the Shelter-in-Place rules?” Given the significant correlation between the 2 variables (77.7% of overall agreement in responses, Cramer *V χ*^2^_1_=0.59; *P*<.001), we used an aggregate outcome: increased e-cigarette use during shelter-in-place. This was a binary variable (yes or no) defined as a self-reported increase in the frequency of e-cigarette use *and* an increase or no change in the nicotine or THC concentration in the e-cigarettes used (Table S1 in Multimedia Appendix 1). Thus, selecting the response “taking more frequent hits or by using more days a month” and also reporting increased strength or no change in the concentration of e-cigarettes were classified as having increased e-cigarette use. Inconsistent changes in the frequency and concentration of e-cigarettes (eg, weaker concentration but more frequent use and vice versa, n=8) were not counted as an increase [55].

Exposure to e-cigarette digital content on social media was coded as a binary variable (yes or no) defined as affirmative responses to the following question: “At any point during Shelter-in-Place have you viewed vape advertisements or vaping digital content on any social media sites?” Both “not sure” and “no” responses were coded as “no exposure.”

### Correlates and Covariates

#### e-Cigarette Use

The survey included images and provided examples of e-cigarette brands in questions about lifetime and past 30-day use of disposable (eg, Puff Bar), pod- or cartridge-based (eg, JUUL), or other types of nicotine e-cigarettes (eg, mod-based e-cigarettes, e-hookahs, and e-cigars) and THC vaporizer products (eg, Evolab).

We assessed e-cigarette dependence using the 4-item e-cigarette dependence scale (EDS) [56], with a possible range from 0 to 16 (Cronbach α=0.87) [57]. The survey also included questions about tobacco use among those who lived with the respondents (eg, a family member or a friend), which we further dichotomized for logistic modeling: any family member or friend versus nobody, the number of people who lived with the respondents (in categories: alone, 1‐2, 3, and ≥4), how many family members and who (eg, a parent and a sibling) were aware that respondents used e-cigarettes (in categories: 0, 1, and ≥2), as well as reasons why respondents reported increased or decreased e-cigarette use during shelter-in-place (eg, being bored, lonely, and stressed).

#### Social Media Use

Participants reported whether they used social media more since shelter-in-place (yes or no) and what types of apps or websites respondents used in the past 30 days; we then derived the number of web-based platforms or apps used by respondents. Social media intensity was measured with 6 survey items (Cronbach α=0.83) adapted from the Facebook Addiction Scale by Andreassen et al [58] rating agreement with statements about social media use on a 5-point Likert scale from 1=never to 5=always; we used the average score similar to past research (Table S2 in Multimedia Appendix 1) [59].

#### COVID-19 and Coping With Shelter-in-Place Orders

Respondents reported whether they had been tested positive for COVID-19 and the methods used to cope with shelter-in-place (eg, using e-cigarettes and social media). We measured anxiety over COVID-19 using agreement with 6 statements on a Likert scale: 1=strongly disagree to 5=strongly agree and calculated the average score of the 6 items (Cronbach α=0.77; Table S2 in Multimedia Appendix 1).

#### Psychological Distress

We used the Kessler 6 Scale to measure shelter-in-place–related psychological distress over the past 30-day recall period (scores ranged from 0 to 24) [60,61]. We summed the score values and classified those with score ≥13 as severe psychological distress during shelter-in-place [60]. Respondents also reported other possible concerns they felt during shelter-in-place and how often they felt lonely (dichotomized to all or most of the time vs less often or never).

#### Sociodemographic Characteristics

The participants provided data about their age (in years); school grade as of fall 2020: high school (grades 9‐12) or middle school (grades 6‐8); self-identified sex; race and ethnicity that were combined to create a four-level covariate representing those who were (1) non-Hispanic African American or Black, (2) non-Hispanic White, (3) non-Hispanic other race, and (4) Hispanic, of any race; as well as mother’s highest level of educational attainment as a proxy for socioeconomic status [27]—a four-level variable: (1) General Education Development test or high school degree or lower, (2) some college, (3) some graduate or professional degree, and (4) unknown.

### Statistical Analysis

All analyses were conducted using SAS software (version 9.4; SAS Institute). Two-sided *P* values ≤.05 were deemed statistically significant. Descriptive statistics included frequencies and proportions for categorical variables, means and SDs or medians and the IQRs (25th and 75th percentiles) for normally and nonnormally distributed continuous variables, respectively. As suggested to be a superior method to handle small sample and sparse data [62], we conducted bivariate and multivariable penalized logistic regressions with profile-likelihood CIs for nonlinear models [63] to assess unadjusted and adjusted odds ratios (AORs and odds ratio) with 95% CIs. In penalized logistic regression modeling, the likelihood is “penalized” by half of the logarithm of the determinant of the information matrix [62].

We assessed adjusted associations of increased e-cigarette use during shelter-in-place with exposure to e-cigarette content on social media and with other predictors of interest that were significant on α=.10 in unadjusted models. We adjusted all multivariable models for potential confounding factors, similar to prior research: age, race and ethnicity, and mother’s educational attainment [27,28,64]. Complete case analysis (n=84) was used in all models because of the small amount of missing data (n=1, 1%). We found no substantial collinearity in the models. Because of the exploratory nature of our study, we report all results that reached statistical significance [65]. We also present supplement models with results significant at *P*≤.007 (ie, .05/7) using Bonferroni correction (Tables S4-S10 in Multimedia Appendix 1).

### Sensitivity Analyses

First, we reran all penalized multivariable logistic regression models while excluding 3 respondents who had reported 0 days and times of e-cigarette use in the past 30 days in the final survey. These data contradicted their prior responses about current e-cigarette use in the screening questionnaire (ie, which violates the eligibility criteria).

Second, we carried out traditional multivariable logistic regression models with normal-based Wald CIs to compare results with the primary analysis that used penalized regression modeling with profile-based CIs. For the third and fourth sensitivity analyses, we carried out multivariable logistic regression models (penalized and traditional for comparison) to assess correlates of 2 separate outcome variables: increased frequency of e-cigarette use and increased concentration of e-cigarettes used.

## Results

### Respondent Characteristics

Most participants were high school students (80/85, 94%), many identified as male (45/85, 53%) and Hispanic (37 of 85, 44%), and the mean age was 16.7 (SD 1.2) years (Table 1). One (1%) respondent was 19 years of age but was still in high school and, thus, was included in the analysis. Many reported that their mothers had at least some college education (37/85, 44%) and had received or were obtaining a graduate or professional degree (18/85, 21%).

**Table 1.** Respondent characteristics (N=85).

Characteristic or behavior	Values^a^
**Demographic characteristics**	
**Age (n=84) (years), mean (SD)**	16.7 (1.2)
**School grade, n (%)**	
High school (9th-12th)	80 (94)
Middle school (6th-8th)	5 (6)
**Self-identified sex, n (%)**	
Female	39 (46)
Male	45 (53)
Other or nonbinary	1 (1)
**Race and ethnicity, n (%)**	
African American or Black and non-Hispanic	13 (15)
Hispanic	37 (44)
White and non-Hispanic	26 (31)
Other race^b^ and non-Hispanic	9 (11)
**Mother’s educational attainment, n (%)**	
GED^c^ or high school or lower	25 (29)
Some college degree	37 (44)
Some graduate or professional degree	18 (21)
Unknown	5 (6)
**e-Cigarette use before and during shelter-in-place orders**	
**Ever-use of e-cigarette products in the lifetime, n (%)**	
Disposable	82 (96)
Pod-based	71 (84)
THC^d^	75 (88)
Other	68 (80)
**e-Cigarette dependence (range 0‐16), median (IQR)**	9 (4-11)
**Change in the frequency of e-cigarette use during shelter-in-place, n (%)**	
More days per month or more hits per day	51 (60)
Fewer days per month or fewer hits per day	28 (33)
No change	6 (7)
**Change in the concentration of e-cigarettes used during shelter-in-place, n (%)**	
Stronger	38 (45)
Weaker	23 (27)
No change	24 (28)
**Tobacco or THC use among coresidents, n (%)**	
Nobody	21 (25)
Family member or friend	64 (75)
Family members only	34 (40)
Friends (nonfamily members) only	22 (26)
Family and Friends	8 (9)
**People you live with, n (%)**	
Alone	3 (4)
1‐2	18 (21)
3	31 (36)
≥4	33 (39)
**Who knows that you use e-cigarettes? n (%)**	
A parent	46 (54)
A sibling	40 (47)
A grandparent	7 (8)
Another relative	15 (18)
No one	12 (14)
**Family members who know you use e-cigarettes, n (%)**	
0	10 (12)
1	49 (58)
≥2	26 (31)
**Social media use**	
**Using social media more since shelter-in-place, n (%)**	83 (98)
**Social Media Intensity score (range 1‐5), median (IQR)**	3.5 (2.7‐3.8)
**Saw e-cigarette digital content on social media, n (%)**	63 (74)
**Apps used in the past 30 days (n=74), n (%)**	
Facebook	42 (57)
Instagram	58 (78)
Snapchat	50 (68)
TikTok	45 (61)
Twitter	44 (59)
WhatsApp	35 (47)
YouTube	51 (69)
Other^e^	4 (5)
**Apps used (n=74, range 1‐8), median (IQR)**	4 (3-6)
**COVID-19 status and coping with shelter-in-place orders**	
**Diagnosed with COVID-19, n (%)**	7 (8)
**Anxiety over COVID-19 (range 1‐5), median (IQR)**	3.8 (3.3‐4.2)
**How are you coping with shelter-in-place? n (%)**	
Being on social media	76 (89)
Facetiming	24 (28)
Streaming videos	29 (34)
Watching television	46 (54)
Playing videogames	48 (56)
Reading	14 (16)
Using e-cigarettes	56 (66)
Drinking alcohol	24 (28)
Having sex	9 (11)
Exercising	27 (32)
Meditating	17 (20)
Other^f^	3 (4)
I am not coping	5 (6)
**Psychological and emotional distress**	
**Feeling lonely all or most of the time, n (%)**	35 (41)
**Psychological distress (n=84)^g^, n (%)**	
Severe (≥13)	39 (46)
Not severe (<13)	45 (53)
**Other concerns endorsed, n (%)**	
Stuck at home with my family all the time	49 (58)
Frustrated that my routine or plan has been disrupted	45 (53)
Not sure when my life will go back to normal	51 (60)
Spending more time on social media	32 (38)
Worried about COVID-19	33 (39)
Not able to meet up or hang out with the people I want to	42 (49)
Angry about the current state of politics	25 (29)
Other^h^	4 (5)

a Results may not add up to 100% or may exceed 100% because of rounding.

b Includes Alaskan Native or American Indian or multiracial, Asian or Native Hawaiian, or Pacific Islander, non-Hispanic.

c GED: General Education Development test.

d THC: tetrahydrocannabinol.

e Includes Among Us, Discord, Teams, and Zoom.

f Includes responses such as “going outside, work, or drugs.”

g One participant had missing values and an unpredictable sum of scores.

h Includes responses such as “having before-lockdown problems, no friends, mental health, or web-based learning is difficult.”

### e-Cigarette Use

All respondents reported past 30-day use of nicotine e-cigarette products, and 68 of 85 (80%) reported past 30-day use of THC vaporizers; 75 of 85 (88%) had ever used THC vapor products. The average and median EDS scores were 8.3 (SD 4.4) and 9 (IQR 4‐11), respectively. Three-quarters of the respondents (64/85, 75%) reported household e-cigarette use among people with whom they lived. Many (51/85, 60%) increased the frequency of e-cigarette use, and the main reasons among 48 (of 51) respondents who reported were (1) being bored (n=32, 67%), (2) stressed (n=27, 56%), (3) lonely (n=21, 44%), and (4) having other people around who used e-cigarettes (n=12, 25%; Table S3 in Multimedia Appendix 1). Many (38/85, 45%) said that e-cigarettes they used were of stronger concentration, and 46 of 85 (54%) reported increased frequency of e-cigarette use of the same or stronger concentration during shelter-in-place (Table S1 in Multimedia Appendix 1).

### Social Media Use

Almost all participants said that they used social media more since shelter-in-place (83/85, 98%), and the intensity of social media use was moderately high (median 3.5 of 5, IQR 2.7‐3.8). Many reported seeing e-cigarette advertisements or other digital content on social media during shelter-in-place (63/85, 74%). Of 85 respondents, 74 (87%) named a total of 11 social media platforms or apps they had been using in the past 30 days (median 4, IQR 3‐6; range 1‐8). Almost all of those 74 reported past-month use of at least 2 social media platforms (n=73, 99%), and 48 (65%) said they used at least 4 platforms. The top 5 web-based platforms listed by the 74 respondents were Instagram (n=58, 78%), YouTube (n=51, 69%), Snapchat (n=50, 68%), TikTok (n=45, 61%), and Twitter (n=44, 59%).

### COVID-19 and Coping With Shelter-in-Place Orders

The level of anxiety over COVID-19 was moderately high among the respondents (median 3.8 of 5, IQR 3.3‐4.2). Participants (N=85) reported 14 ways how they coped with shelter-in-place; the top 5 were examined in subsequent regression analyses: being on social media (n=76, 89%), using e-cigarettes (n=56, 66%), playing videogames (n=48, 56%), watching television (n=46, 54%), and streaming videos (n=29, 34%).

### Psychological and Emotional Distress

Many participants reported feeling lonely (35/85, 41%), and the average level of psychological distress was 12 (SD 5.1), with 46% (39/84) reporting severe psychological distress. Participants (N=85) also endorsed the following concerns: not being sure when life would go back to normal (n=49, 58%), being “stuck at home” with their family all the time (n=49, 58%), being frustrated that their routine or plan has been disrupted (n=45, 53%), being unable to meet up or hang out with the people they wanted to (n=42, 49%), and being worried about the COVID-19 (n=33, 39%).

### Correlates of Increased e-Cigarette Use

**Figure 1.**
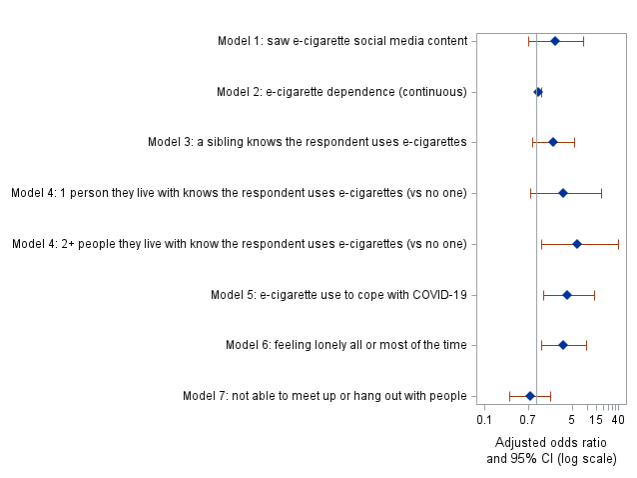
Forest plot based on results of 7 adjusted penalized logistic regressions with self-reported increased adolescent e-cigarette use during shelter-in-place as the outcome (n=84). Results shown are adjusted odds ratios and their 95% confidence intervals (CIs) for the main predictors of interest in each of the 7 models assessed. All models were adjusted for age, race and ethnicity, and mother’s highest level of educational attainment.

The association between increased e-cigarette use during shelter-in-place and exposure to e-cigarette–related digital content on social media was borderline significant (odds ratio 2.58, 95% CI 0.98‐7.13; *P*=.06) in the unadjusted analysis (Table 2) and nonsignificant (AOR 2.34, 95% CI 0.71‐8.46; *P*=.19) after controlling for demographics (Figure 1 and Table S4 in Multimedia Appendix 1). Among the other 6 predictors assessed in the adjusted modeling (Figure 1 and Table S4 in Multimedia Appendix 1), 3 were positively associated with increased e-cigarette use: having ≥2 family members (vs no one) who were aware about participants’ e-cigarette use (AOR 6.42, 95% CI 1.29‐39.49; *P*=.04), using e-cigarettes to cope with shelter-in-place (AOR 4.06, 95% CI 1.39‐13.41; *P*=.02), and feeling lonely (AOR 3.33, 95% CI 1.27‐9.42; *P*=.02). Older participants were more likely to report increased e-cigarette use based on all models (Table S4 in Multimedia Appendix 1).

**Table 2.** Unadjusted associations of increased e-cigarette use with demographic and behavioral characteristics: results from unadjusted penalized logistic regression models (N=85).

Variable (responses)	Increased (n=46)	Did not increase (n=39)	OR^a^ (95% CI)	*P* value
**Demographic characteristics**				
**Age (n=84) (years), mean (SD)**	17.1 (1.2)	16.17 (1.1)	2.03 (1.35‐3.31)	.002
**In high school (9th-12th), n (%)**	45 (98)	35 (90)	3.85 (0.67‐39.62)	.20
** Self-identified sex, n (%)**				
Female	24 (52)	15 (38)	1.80 (0.77-4.32)	.19
Male	21 (46)	24 (61)	Reference	—^b^
Other or nonbinary^c^	1 (2)	0 (0)	—	—
** Race and ethnicity, n (%)**				
African American or Black and non-Hispanic	10 (22)	3 (8)	1.91 (0.48‐9.03)	.39
Hispanic	15 (33)	22 (56)	0.44 (0.16‐1.19)	.12
White and non-Hispanic	16 (35)	10 (26)	Reference	—
Another race^d^ and non-Hispanic	5 (6)	4 (10)	0.78 (0.18‐3.52)	.75
** Mother’s educational attainment, n (%)**				
GED^e^ or high school or lower	12 (26)	13 (33)	0.60 (0.18‐1.99)	.42
Some college degree	21 (46)	16 (41)	0.85 (0.27‐2.59)	.78
Some graduate or professional degree	11 (24)	7 (18)	Reference	—
Unknown	2 (4)	3 (8)	0.47 (0.06‐3.00)	.46
**e-Cigarette use**				
**e-Cigarette dependence (range 0‐16), median (IQR)**	10 (7‐11)	7 (3-11)	1.12 (1.02‐1.25)	.03
** Who knows that you use e-cigarette? n (%)**				
A parent	28 (61)	18 (46)	1.79 (0.77‐4.25)	.19
A sibling	26 (57)	14 (36)	2.27 (0.97‐5.49)	.07
A grandparent	4 (9)	3 (8)	1.10 (0.25‐5.23)	.90
Another relative	9 (20)	6 (15)	1.31 (0.44‐4.10)	.64
No one	4 (9)	8 (21)	0.39 (0.11‐1.30)	.15
** Family members who know you use e-cigarettes, n (%)**				
0	3 (7)	7 (18)	Reference	—
1	26 (57)	23 (59)	2.42 (0.64‐10.93)	.23
≥2	17 (37)	9 (23)	3.95 (0.93‐19.71)	.08
** Tobacco or THC**^f^ **use among people with whom you currently live, n (%)**				
Family member or friend	36 (78)	28 (72)	1.40 (0.53‐3.75)	.50
Nobody	10 (22)	11 (28)	Reference	—
** People you live with, n (%)**				
Alone	3 (7)	0 (0)	4.56 (0.36‐648.0)	.40
1‐2	11 (24)	7 (18)	Reference	—
3	15 (33)	16 (41)	0.61 (0.19‐1.92)	.42
4+	17 (37)	16 (41)	0.69 (0.22‐2.14)	.54
**COVID-19–related factors**				
**Diagnosed with COVID-19, n (%)**	2 (4)	5 (13)	0.35 (0.06‐1.56)	.22
**Anxiety over COVID-19 (range 1‐5), median (IQR)**	3.9 (3.5‐4.2)	3.8 (3.3‐4.2)	1.07 (0.62‐1.87)	.81
**Strongly willing to be vaccinated against the COVID-19 infection, n (%)**	35 (76)	26 (67)	1.57 (0.62‐4.06)	.35
** How are you coping with shelter-in-place? n (%)**				
Being on social media	42 (91)	34 (87)	1.51 (0.40‐6.01)	.56
Streaming videos	18 (39)	11 (28)	1.61 (0.66‐4.04)	.31
Watching television	6 (57)	20 (51)	1.23 (0.53‐2.88)	.64
Playing videogames	29 (63)	19 (49)	1.77 (0.76‐4.22)	.20
Using e-cigarettes	36 (78)	20 (51)	3.31 (1.34‐8.59)	.01
**Social media**				
**Social Media Intensity (range 1‐5), median (IQR)**	3.5 (2.7‐3.8)	3.3 (2.3‐3.8)	1.22 (0.77‐1.96)	.40
**Using social media more since shelter-in-place, n (%)**	45 (98)	38 (97)	0.85 (0.07‐10.74)	.91
**Saw e-cigarette digital content on social media, n (%)**	38 (83)	25 (64)	2.58 (0.98‐7.13)	.06
** Apps used in the past 30 days (n=74), n (%)**				
TikTok	25 (66)	20 (56)	1.52 (0.61‐3.88)	.38
Instagram	30 (79)	28 (78)	1.07 (0.36‐3.19)	.91
Facebook	23 (61)	19 (53)	1.36 (0.55‐3.41)	.51
Twitter	25 (66)	19 (53)	1.70 (0.68‐4.33)	.27
Snapchat	25 (66)	25 (69)	0.85 (0.32‐2.22)	.75
WhatsApp	21 (55)	14 (39)	1.91 (0.77‐4.83)	.17
YouTube	24 (63)	27 (75)	0.58 (0.21‐1.54)	.29
Other^g^	2 (4)	2 (5)	0.95 (0.14‐6.44)	.96
Apps used in the past 30 days (n=74), median (IQR)	4 (3-7)	4 (3-5)	1.12 (0.86‐1.46)	.41
**Emotional and psychological distress**				
**Feeling lonely all or most of the time, n (%)**	18 (50)	9 (26)	4.15 (1.68‐10.91)	.003
** Psychological distress (n=84),**^h^ **n (%)**				
Severe (13+)	17 (47)	15 (43)	1.23 (0.53‐2.90)	.64
No severe psychological distress (<13)	19 (53)	20 (57)	Reference	—
** Other concerns endorsed, n (%)**				
Stuck at home with my family all the time	27 (59)	22 (56)	1.10 (0.47‐2.58)	.83
Frustrated that my routine or plan has been disrupted	28 (61)	17 (44)	1.98 (0.85‐4.73)	.12
Not sure when my life will go back to normal	28 (61)	23 (59)	1.08 (0.46‐2.56)	.86
Spending more time on social media	23 (50)	9 (23)	1.25 (0.53‐3.00)	.62
Worried about COVID-19	19 (41)	14 (36)	0.73 (0.31‐1.69)	.46
Not able to meet up or hang out with people	21 (46)	21 (54)	3.21 (1.30‐8.40)	.02
Angry about the current state of politics	16 (35)	9 (23)	1.74 (0.69‐4.59)	.26

a OR: odds ratio.

b Not applicable.

c Excluded from logistic regression.

d Alaskan Native or American Indian or multiracial, Asian or Native Hawaiian, or Pacific Islander, non-Hispanic

e GED: General Education Development test.

f THC: tetrahydrocannabinol.

g Includes the following: Among Us, Discord, Teams, and Zoom.

h One participant was excluded due to missing values and an unpredictable sum of scores.

### Sensitivity Analyses

Results of the first sensitivity analysis (Table S5 in Multimedia Appendix 1), while excluding 3 observations with inconsistent data on past 30-day e-cigarette use (n=81), confirmed our primary findings. Results from the second sensitivity analysis to compare traditional versus penalized logistic regression models showed similar findings in terms of the directionality and the significance of the associations assessed. The only exception was a statistically significant association in the traditional logistic regression for increased use and e-cigarette use dependence (AOR 1.13, 95% CI 1.01‐1.28; *P*=.04; Table S6 in Multimedia Appendix 1), whereas this result had borderline significance in the primary analysis (AOR 1.12, 95% CI 1.00‐1.25; *P*=.06; Table S4 in Multimedia Appendix 1).

The third sensitivity analysis of correlates of increased frequency of use revealed consistent results with the primary findings for increased e-cigarette use (Tables S7 and S8 in Multimedia Appendix 1). The fourth sensitivity analysis of correlates of increased concentration of e-cigarettes used also showed similar results, except for no or borderline significant associations with increased age in both penalized and traditional logistic regressions, and consistently positive associations with increased e-cigarette use dependence and lower odds for increased concentration among those who were limited in social interaction during shelter-in-place (Tables S9 and S10 in Multimedia Appendix 1).

## Discussion

### Principal Findings

This cross-sectional study of 85 California adolescents using e-cigarettes revealed many increased social media use during shelter-in-place (83/85, 98%), often as a way of coping with shelter-in-place (76/85, 89%). We found that a larger proportion of respondents who reported increased e-cigarette use (vs those who did not) also viewed e-cigarette–related social media digital content during shelter-in-place (38/46, 82% vs 25/39, 64%), but differences were not statistically significant likely due to the small sample size. Comparable to prior research [66,67], the average EDS score in our sample was 8.3 (SD 4.4). Consistent with national [8,21,25] and California surveys [68], and in accordance with previous studies [46,49,50,69], older adolescents, those who used e-cigarettes to cope with shelter-in-place, and those who had ≥2 family members being aware of participants’ e-cigarette use were more likely to report changes of increased e-cigarette use during shelter-in-place. A novel finding of our study was that loneliness was associated with increased e-cigarette use, in contrast with prior studies that found no significant differences [70] or associations with decreased e-cigarette use [71] among young people during the COVID-19 pandemic. We found only one prepandemic study suggesting a higher risk of e-cigarette use initiation among tobacco-naïve adolescents with high internalizing problems, including loneliness [49].

### Comparison With Prior Work

Our hypothesis that the exposure to e-cigarette–related digital content would impact adolescent e-cigarette use during shelter-in-place was based on previous studies, which were mainly focused on tobacco-naïve youths [36,38], compared tobacco users to nonusers [33,72], or were conducted before the COVID-19 pandemic. In contrast, our survey was done during the unique time and settings of shelter-in-place orders and remote schooling, and the sample was restricted to adolescents who were currently using e-cigarettes. However, we lacked data on the type of advertisement or digital content that the respondents had seen on social media. Being exposed to both pro- and anti–e-cigarette use digital content [73] could have shifted the results toward the null. These findings warrant future research with a larger sample to better understand whether different types of exposure to e-cigarette content on social media may have different impacts on current consumers of e-cigarettes or on tobacco-naïve youths, including after the COVID-19 pandemic.

The risk of adolescent e-cigarette use can vary by the type and frequency of web-based venues used [36]. Camenga et al [38] found that Facebook advertisements increased cigarette use in the cohort of e-cigarette–naïve youths. Exposure to cannabis advertisements on Facebook, Instagram, and Twitter was also associated with increased past-year cannabis use among adolescents [43]. Unlike past research before the COVID-19 pandemic, we did not see any differences in the odds of increased e-cigarette use associated with the use of specific social media platforms during shelter-in-place. This could likely be explained by increased overall social media use among adolescents during shelter-in-place and a high proportion of respondents who used multiple existing social media platforms (up to 8 web-based platforms or apps) in our study. Further, our survey rather asked about the use of web-based platforms in general and not about exposure to e-cigarette–related content viewed on specific social media sites.

By providing trustworthy and relevant content [74], social media can become an effective channel for the implementation and promotion of intervention measures to prevent both cannabis and nicotine e-cigarette use and couse among adolescents. Although data to support the effectiveness of web-based e-cigarette cessation interventions among adolescents are lacking [75,76], past research has shown feasibility [77,78] and successes [79] of web-based interventions for smoking prevention among adolescents and young adults and viability of recruitment of young adults through social media in e-cigarette use cessation [80]. However, given the increased social media use among young people in recent years and especially during the COVID-19 pandemic, improved regulations are needed to make social media use safer for adolescents [81]. In addition to the protection of personal data, such regulations should also be designed to prevent abilities of advertisers to use social media algorithms and marketing strategies for manipulating adolescent users into viewing e-cigarette advertisement or other pro–e-cigarette content [82].

Secondhand smoke exposure among family or friends and a pro–e-cigarette social environment can increase the risk for e-cigarette use initiation [46] and susceptibility to both cigarette [48,69] and e-cigarette use [47,48]. Despite not being statistically associated with increased e-cigarette use during shelter-in-place, 64 of 85 (75%) of our respondents reported household e-cigarette use among their coresidents; and those who increased e-cigarette use were significantly more likely to report 2 or more family members who were aware of participants’ e-cigarette use. These findings raise questions regarding potential approval or indifference and lack of concern toward adolescent e-cigarette use among family members and their nearest social environment. Future research is recommended to assess attitudes and perceived harms among relatives or coresidents of adolescents and its impact on e-cigarette use.

Mental health problems and psychological distress may increase the risk for e-cigarette use initiation [49,50] and current use [12,51,52]. The elevated risk of adolescent e-cigarette use has been associated with internalizing (eg, feeling lonely and depressed) and externalizing (eg, conduct disorder) problems [49,51] and perceived stress [51]. The prevalence of mental health conditions among adolescents during the lockdown and social isolation period of the COVID-19 pandemic accelerated the youth mental health crisis [83,84], resulting in increased rates of anxiety and depressive symptoms [52,85] and suspected suicidal attempts [86]. A survey of Utah youths showed an increase in psychological distress indicators before versus after the COVID-19 pandemic period from an average level of 7.8 to 8.8 and revealed a positive association between psychological distress and e-cigarette use [52]. Compared to the Utah study, the average level of psychological distress among our respondents was higher (mean 12, SD 5.1), suggesting that many adolescents were experiencing at least moderate mental distress [87]; over 45% screened for severe psychological distress (39/84, 46.4%). Unlike the Utah survey that included both tobacco users and nonusers [52], our study restricted to e-cigarette users found that no differences of reporting increased e-cigarette use during shelter-in-place by the level of psychological distress [49,50].

Another novel aspect of the study is the measurement of cannabis vaporizer use during shelter-in-place. Earlier studies among US youths have found that 30.6% of those who had ever used e-cigarettes reported THC and nicotine couse [88]. In our survey, 88% (75/85) reported ever-use and 80% (68/85) reported past 30-day use of THC vaporizers in addition to nicotine e-cigarettes, highlighting a high prevalence of couse among those who had used nicotine e-cigarettes in the past month. Our study included adolescents residing in California, which was the first state to legalize medical cannabis in 1996 and adult recreational cannabis sales since 2018 [89], although some authors claim no effect of such regulations on adolescent use [90]. Concerns regarding mental health and the increased risk of psychological distress during shelter-in-place among adolescents using both substances underscore the need for further studies of tobacco and cannabis couse among adolescents with mental health symptoms [12,52].

### Limitations

First, this survey had a descriptive, cross-sectional study design; causal inferences may not be derived. However, several key features of the survey design, including eligibility criteria, the time frame, and the language used in the questionnaire, have the potential to directly demonstrate the impact of shelter-in-place on the associations assessed. Second, the potential for generalizability of the study results outside California may be limited, as this was a sample of California adolescents only. However, the sample matches the demographic characteristics of California, with 44% (37/85) of our respondents identified as Hispanic, which aligns with prior California surveys in 2018 [68] and 2020 [91] that reported 47% and 52% participants of Hispanic ethnicity, respectively. Third, due to the small sample size, statistical power to detect significant differences was limited. Fourth, these were self-reported data, collected through web-based questionnaires during the COVID-19 lockdown and remote schooling, when adolescents could have been more closely monitored by their parents or guardians. Fifth, the survey did not ask about whether the respondents were aware of e-cigarette, or vaping product, use-associated lung injury. The increased public awareness regarding the harmful health effects of e-cigarettes associated with the 2019 e-cigarette, or vaping product, use-associated lung injury outbreak [92] might have contributed to the 2020‐2021 decrease in the youth e-cigarette use [26].

### Conclusions

Participants exposed to e-cigarette digital content had twice the odds of reporting increased e-cigarette use during shelter-in-place, but the results were not statistically significant in the adjusted analysis. Given almost all participants reported using social media more during shelter-in-place and associations of increased e-cigarette use with loneliness and coping with shelter-in-place, future e-cigarette use interventions should consider leveraging of digital platforms while addressing the mental health consequences of the COVID-19 pandemic.

## Supplementary material

10.2196/49779 Multimedia Appendix 1 Supplementary information.

## Abbreviations

AOR: adjusted odds ratio
EDS: e-cigarette dependency scale
THC: tetrahydrocannabinol
