# Supplementary material for: Social Media Exposure and Other Correlates of Increased e-Cigarette Use Among Adolescents During Remote Schooling: Cross-Sectional Study
Source: JMIR Pediatr Parent. 2024 Oct 21;7:e49779. doi: 10.2196/49779 (PMC11515927; doi:10.2196/49779)
Supplement: Multimedia Appendix 1 [file pediatrics-v7-e49779-s001.docx]

# **Table S1.** Outcome categorization and frequency (N=85)

| **Concentration/Frequency** | **Frequency (%)** | **E-cigarette Change** | **Increased E-cigarette Use (Outcome)** |
| --- | --- | --- | --- |
| Stronger/More | 35 (41.2) | Increased  (n=46, 54.1%) | Yes  (n=46, 54.1%) |
| Same strength/More | 11 (12.9) |  |  |
| Weaker/Less | 16 (18.8) | Decreased  (n=27, 31.8%) | No  (n=39, 45.9%) |
| Weaker/Same | 2 (2.4) |  |  |
| Same strength/Less | 9 (10.6) |  |  |
| No change | 4 (4.7) | No Change  (n=4, 4.7%) |  |
| Stronger/Less | 3 (3.5) | Inconsistency^a^  (n=8, 9.4%) |  |
| Weaker/More | 5 (5.9) |  |  |

^a^We deemed inconsistency as no increase in e-cigarette use assuming that the amount of nicotine/tetrahydrocannabinol (THC) remained the same

# **Table S2.** Survey items used to assess Social Media Intensity and Anxiety Over COVID-19 scales

| **Scale** | **Items** | **Cronbach’s α** |
| --- | --- | --- |
| Social Media Intensity^a^ | (1) “I spend a lot of time thinking about social media or planning how to use it”;  (2) “I feel an urge to use social media more and more”;  (3) “I use social media in order to forget about personal problems”;  (4) “I have tried to cut down on the use of social media without success”;  (5) “I become restless or troubled if I have been prohibited from using social media”;  (6) “I use social media so much that it has had a negative impact on my studies/job.” | .83 |
| Anxiety Over COVID-19 | (1) “I am worried about catching coronavirus”;  (2) “I am worried about a family member catching coronavirus”;  (3) “Vaping increases the risk of catching coronavirus”;  (4) “Vaping increases the risk of having a more severe case of coronavirus infection”;  (5) “I wear a face mask when I go out in public”;  (6) “I am avoiding large social gatherings.” | .77 |

^a^Adapted from the Facebook Addiction Scale by Andreassen et al., 2012

# **Table S3.** Reasons for change in e-cigarette frequency during Shelter-in-Place Orders

| **Why did you increase your use? (n=48)** | **N (%)** |
| --- | --- |
| Because I am bored | 32 (66.7) |
| Because I am stressed | 27 (56.3) |
| Because I am lonely | 21 (43.8) |
| Because there are more people around me who vape | 12 (25.0) |
| Other^a^ | 3 (6.3) |
| **Why did you decrease your use? (n=18)** |  |
| Because I am home, and I am worried my family will get to know | 10 (55.6) |
| Because I can’t get my products as easily | 5 (27.8) |
| Because I am worried about my health | 11 (61.1) |
| Because I am worried about catching coronavirus | 4 (22.2) |
| Because I am having fewer social interactions with my friends | 4 (22.2) |

^a”^I am curious/want to try when offered; depression and anxiety; it is fun and gives me and my friends something to do/look forward to.”

# **Table S4.** Multivariable analysis of associated factors with the increased e-cigarette use during Shelter-in-Place orders: Results from *penalized* logistic regression models adjusted for demographics (N=84)

| **Characteristic** | **Model 1** | **Model 2** | **Model 3** | **Model 4** | **Model 5** | **Model 6** | **Model 7** |
| --- | --- | --- | --- | --- | --- | --- | --- |
| **Sociodemographic characteristics / Main predictor of interest** | AOR (95% CI)^a^,  *p*-value | | | | | | |
| Age | 1.79 (1.16-2.93), *p*=.02* | 1.81 (1.18-2.96), *p*=.01* | 1.86 (1.22-3.05), *p*=.009* | 1.89 (1.24-3.13), *p*=.008* | 2.12 (1.33-3.72), *p*=.004** | 1.81 (1.17-3.01), *p*=.01* | 1.82 (1.21-2.93), *p*=.01* |
| **Race-ethnicity** |  |  |  |  |  |  |  |
| African American/Black, non-Hispanic | 1.07 (0.21-6.01), *p*=.94 | 1.57 (0.34-8.44),  *p*=.59 | 1.53 (0.33-8.29), *p*=.61 | 1.32 (0.25-7.50), *p*=.76 | 1.23 (0.22-7.55), *p*=.81 | 1.26 (0.25-7.21), *p*=.79 | 1.50 (0.33-7.76), *p*=.62 |
| Hispanic, any race | 0.46 (0.13-1.57), *p*=.24 | 0.49 (0.14-1.60), *p*=.26 | 0.67 (0.21-2.16), *p*=.51 | 0.47 (0.14-1.58), *p*=.25 | 0.50 (0.15-1.64), *p*=.27 | 0.63 (0.19-2.11), *p*=.47 | 0.60 (0.19-1.90), *p*=.40 |
| Other | 0.53 (0.10-2.70), *p*=.46 | 0.71 (0.14-3.57), *p*=.69 | 0.67 (0.14-3.37), *p*=.64 | 0.52 (0.10-2.71), *p*=.45 | 0.55 (0.11-2.77), *p*=.49 | 0.62 (0.12-0.12), *p*=.57 | 0.72 (0.15-3.46), *p*=.69 |
| White, non-Hispanic | Reference | Reference | Reference | Reference | Reference | Reference | Reference |
| **Mother’s educational attainment** |  |  |  |  |  |  |  |
| (Some) College degree | 1.04 (0.30-3.50), *p*=.95 | 1.07 (0.31-3.68), *p*=.92 | 0.86 (0.24-2.96), *p*=.82 | 0.68 (0.17-2.48), *p*=.59 | 0.91 (0.25-3.19), *p*=.88 | 0.89 (0.23-3.29),  p=.86 | 1.04 (0.30-3.51), *p*=.96 |
| GED^c^/High School or below | 1.30 (0.33-5.37), *p*=.72 | 1.42 (0.34-6.24), *p*=.64 | 1.05 (0.26-4.36), *p*=.95 | 0.79 (0.17-3.46), *p*=.76 | 1.90 (0.44-9.04), *p*=.42 | 1.06 (0.25-4.65), *p*=.94 | 1.20 (0.30-4.87),  *p*=.81 |
| Unknown | 1.51 (0.16-15.11), *p*=.74 | 1.31 (0.15-11.44), *p*=.82 | 0.67 (0.08-4.99), *p*=.72 | 0.82 (0.10-6.28), *p*=.86 | 0.96 (0.11-8.06), *p*=.97 | 0.52 (0.06-4.42), *p*=.58 | 0.82 (0.10-6.19),  *p*=.86 |
| (Some) Graduate or professional degree | Reference | Reference | Reference | Reference | Reference | Reference | Reference |
| Saw e-cigarette social media content | 2.34 (0.71-8.46), *p*=.19 | N/A | N/A | N/A | N/A | N/A | N/A |
| E-cigarette dependence | N/A | 1.12 (1.00-1.25), *p*=.06 | N/A | N/A | N/A | N/A | N/A |
| A sibling knows the respondent uses e-cigarettes | N/A | N/A | 2.13 (0.85-5.56), *p*=.13 | N/A | N/A | N/A | N/A |
| **No of family members who know you use e-cigarettes** |  |  |  |  |  |  |  |
| No one | Reference | Reference | Reference | Reference | Reference | Reference | Reference |
| One person | N/A | N/A | N/A | 3.47 (0.77-19.08), *p*=.14 | N/A | N/A | N/A |
| Two or more | N/A | N/A | N/A | 6.42 (1.29-39.49), *p*=.04* | N/A | N/A | N/A |
| E-cigarette use to cope with COVID-19 | N/A | N/A | N/A | N/A | 4.06 (1.39-13.41), *p*=.02* | N/A | N/A |
| Feeling lonely all or most of the time | N/A | N/A | N/A | N/A | N/A | 3.33 (1.27-9.42), *p*=.02* | N/A |
| Not able to meet up/hang out with the people | N/A | N/A | N/A | N/A | N/A | N/A | 0.77 (0.30-1.92), *p*=.58 |

Notes: Primary analysis – increased e-cigarette use defined as increased frequency and an increase or no change in the concentration of e-cigarettes used.

^a^AOR: Adjusted odds ratio; CI: confidence interval

^b^GED: General Education Development Test

^c^N/A: Not applicable

*– Indicates statistically significant results (p≤.05); ** – Bonferroni significance level to account for carrying out seven models (0.05/7): p≤.007

# **Table S5.** Multivariable analysis of associated factors with the increased e-cigarette use during Shelter-in-Place orders: Results from *penalized* logistic regression models adjusted for demographics (N=81)

| **Characteristic** | **Model 1** | **Model 2** | **Model 3** | **Model 4** | **Model 5** | **Model 6** | **Model 7** |
| --- | --- | --- | --- | --- | --- | --- | --- |
| **Sociodemographic characteristics / Main predictor of interest** | AOR (95% CI)^a^,  *p*-value | | | | | | |
| Age | 1.82 (1.16-3.06), *p*=.02* | 1.82 (1.18-3.03), p=.01* | 1.85 (1.20-3.07), p=.01* | 1.91 (1.22-3.20), p=.01* | 2.11 (1.31-3.72), p=.006** | 1.82 (1.16-3.08), p=.02* | 1.82 (1.19-3.00), p=.01* |
| **Race-ethnicity** |  |  |  |  |  |  |  |
| African American/Black, non-Hispanic | 0.88 (0.16-5.14), p=.89 | 1.44 (0.31-7.71), p=.67 | 1.4 (0.30-7.57), p=.69 | 1.12 (0.21-6.52), p=.90 | 1.16 (0.21-6.98), p=.87 | 1.15 (0.23-6.66), p=.87 | 1.31 (0.28-6.91), p=.74 |
| Hispanic, any race | 0.39 (0.10-1.37), p=.17 | 0.46 (0.13-1.53), p=.23 | 0.61 (0.19-1.99), p=.43 | 0.42 (0.12-1.41), p=.18 | 0.47 (0.14-1.56), p=.24 | 0.58 (0.17-1.94), p=.39 | 0.53 (0.16-1.72), p=.31 |
| Other | 0.43 (0.08-2.24), p=.34 | 0.64 (0.13-3.22), p=.61 | 0.59 (0.12-2.97), p=.54 | 0.42 (0.08-2.25), p=.33 | 0.52 (0.10-2.59), p=.44 | 0.56 (0.11-2.82), p=.49 | 0.61 (0.13-2.96), p=.55 |
| White, non-Hispanic | Reference | Reference | Reference | Reference | Reference | Reference | Reference |
| **Mother’s educational attainment** |  |  |  |  |  |  |  |
| (Some) College degree | 1.13 (0.33-3.88), p=.85 | 1.12 (0.32-3.85), p=.86 | 0.93 (0.26-3.21), p=.92 | 0.72 (0.18-2.66), p=.65 | 0.96 (0.27-3.38), p=.96 | 0.95 (0.25-3.52), p=.94 | 1.14 (0.33-3.91), p=.84 |
| GED^c^/High School or below | 1.37 (0.34-5.73), p=.67 | 1.42 (0.35-6.20), p=.64 | 1.09 (0.27-4.52), p=.91 | 0.78 (0.17-3.47), p=.76 | 1.86 (0.43-8.77), p=.43 | 1.11 (0.26-4.83), p= 0.90 | 1.26 (0.32-5.20), p=.75 |
| Unknown | 2.01 (0.19-23.7), p=.59 | 1.38 (0.15-12.81), p=.79 | 0.89 (0.10-7.71), p=.92 | 1.03 (0.11-9.43), p=.98 | 1.02 (0.11-9.03), p=.99 | 0.60 (0.06-5.64), p=.68 | 0.97 (0.11-8.63), p=.98 |
| (Some) Graduate or professional degree | Reference | Reference | Reference | Reference | Reference | Reference | Reference |
| Saw e-cigarette social media content | 2.67 (0.78-10.36), *p*=.15 | N/A | N/A | N/A | N/A | N/A | N/A |
| E-cigarette dependence | N/A | 1.10 (0.99-1.24), p=.12 | N/A | N/A | N/A | N/A | N/A |
| A sibling knows the respondent uses e-cigarettes | N/A | N/A | 2.02 (0.79-5.34), p=.16 | N/A | N/A | N/A | N/A |
| **No of family members who know you use e-cigarettes** |  |  |  |  |  |  |  |
| No one | Reference | Reference | Reference | Reference | Reference | Reference | Reference |
| One person | N/A | N/A | N/A | 4.18 (0.90-23.81), p=.10 | N/A | N/A | N/A |
| Two or more | N/A | N/A | N/A | 6.89 (1.36-43.6), p=.04* | N/A | N/A | N/A |
| E-cigarette use to cope with COVID-19 | N/A | N/A | N/A | N/A | 3.57 (1.20-11.98), p=.03* | N/A | N/A |
| Feeling lonely all or most of the time | N/A | N/A | N/A | N/A | N/A | 3.08 (1.16-8.81), p=.03* | N/A |
| Not able to meet up/hang out with the people | N/A | N/A | N/A | N/A | N/A | N/A | 0.69 (0.26-1.78), p=.47 |

Notes: Sensitivity analysis 1 – increased e-cigarette use defined as increased frequency and an increase or no change in the concentration of e-cigarettes used. Three respondents, who had reported 0 days and times of e-cigarette use in the past 30 days in the final survey, were excluded. These data contradicted their prior responses about current e-cigarette use in the screening questionnaire (i.e., which violates the eligibility criteria).

aAOR: Adjusted odds ratio; CI: confidence interval

bGED: General Education Development Test

cN/A: Not applicable

*– Indicates statistically significant results (p≤.05); ** – Bonferroni significance level to account for carrying out seven models (.05/7): p≤.007

# **Table S6.** Multivariable analysis of associated factors with the increased e-cigarette use during Shelter-in-Place orders: Results from *traditional* logistic regression models adjusted for demographics (N=84)

| **Characteristic** | **Model 1** | **Model 2** | **Model 3** | **Model 4** | **Model 5** | **Model 6** | **Model 7** |
| --- | --- | --- | --- | --- | --- | --- | --- |
| **Sociodemographic characteristics / Main predictor of interest** | AOR (95% CI)^a^,  *p*-value | | | | | | |
| Age | 1.95 (1.19-3.19), p=.008* | 1.98 (1.20-3.24) p=.007** | 2.04 (1.25-3.34), p=.004** | 2.10 (1.27-3.47), p=.004** | 2.41 (1.38-4.20), p=.002** | 1.98 (1.20-3.27), p=.008* | 1.98 (1.23-3.18), p=.005** |
| **Race-ethnicity** |  |  |  |  |  |  |  |
| African American/Black, non-Hispanic | 1.10 (0.18-6.53), p=.92 | 1.74 (0.31-9.66), p=.53 | 1.70 (0.31-9.42), p=.55 | 1.40 (0.23-8.61), p=.72 | 1.33 (0.20-8.73), p=.77 | 1.35 (0.23-8.02), p=.74 | 1.65 (0.31-8.72), p=.56 |
| Hispanic, any race | 0.41 (0.11-1.57), p=.20 | 0.44 (0.12-1.59), p=.21 | 0.65 (0.19-2.24), p=.50 | 0.43 (0.12-1.58), p=.21 | 0.46 (0.13-1.63), p=.23 | 0.61 (0.17-2.18), p=.45 | 0.58 (0.17-1.94), p=.37 |
| Other | 0.50 (0.09-2.81), p=.43 | 0.70 (0.13-3.88), p=.69 | 0.66 (0.12-3.57), p=.63 | 0.48 (0.08-2.80), p=.42 | 0.52 (0.09-2.91), p=.46 | 0.59 (0.11-3.26), p=.54 | 0.71 (0.14-3.69), p=.68 |
| White, non-Hispanic | Reference | Reference | Reference | Reference | Reference | Reference | Reference |
| **Mother’s educational attainment** |  |  |  |  |  |  |  |
| (Some) College degree | 1.03 (0.28-3.74), p=.97 | 1.07 (0.29-3.97), p=.92 | 0.83 (0.22-3.09), p=.78 | 0.62 (0.15-2.61), p=.52 | 0.87 (0.23-3.35), p=.84 | 0.87 (0.21-3.53), p=.84 | 1.03 (0.28-3.75), p=.96 |
| GED^c^/High School or below | 1.34 (0.31-5.88), p=.69 | 1.52 (0.33-7.03), p=.59 | 1.05 (0.23-4.68), p=.95 | 0.75 (0.15-3.72), p=.72 | 2.13 (0.43-10.68), p=.36 | 1.07 (0.23-5.01), p=.94 | 1.23 (0.28-5.32), p=.78 |
| Unknown | 1.57 (0.13-18.35), p=.72 | 1.33 (0.13-13.76), p=.81 | 0.60 (0.07-5.50), p=.65 | 0.74 (0.08-7.24), p=.80 | 0.92 (0.09-9.29), p=.94 | 0.45 (0.04-4.80), p=.51 | 0.76 (0.08-7.00), p=.80 |
| (Some) Graduate or professional degree | Reference | Reference | Reference | Reference | Reference | Reference | Reference |
| Saw e-cigarette social media content | 2.66 (0.71-9.89), p=.14 | N/A | N/A | N/A | N/A | N/A | N/A |
| E-cigarette dependence | N/A | 1.13 (1.01-1.28), p=.04* | N/A | N/A | N/A | N/A | N/A |
| A sibling knows the respondent uses e-cigarettes | N/A | N/A | 2.35 (0.87-6.35), p=.09 | N/A | N/A | N/A | N/A |
| **No of family members who know you use e-cigarettes** |  |  |  |  |  |  |  |
| No one | Reference | Reference | Reference | Reference | Reference | Reference | Reference |
| One person | N/A | N/A | N/A | 4.38 (0.78-24.58), p=.09 | N/A | N/A | N/A |
| Two or more | N/A | N/A | N/A | 8.87 (1.40-56.14), p=.02* | N/A | N/A | N/A |
| E-cigarette use to cope with COVID-19 | N/A | N/A | N/A | N/A | 5.09 (1.51-17.15), p=.009* | N/A | N/A |
| Feeling lonely all or most of the time | N/A | N/A | N/A | N/A | N/A | 3.95 (1.36-11.49), p=.01* | N/A |
| Not able to meet up/hang out with the people | N/A | N/A | N/A | N/A | N/A | N/A | 0.74 (0.28-1.96), p=.54 |

Notes: Sensitivity analysis 2 – increased e-cigarette use defined as increased frequency and an increase or no change in the concentration of e-cigarettes used.

^a^AOR: Adjusted odds ratio; CI: confidence interval

^b^GED: General Education Development Test

^c^N/A: Not applicable

*– Indicates statistically significant results (p≤.05); ** – Bonferroni significance level to account for carrying out seven models (.05/7): p≤.007

# **Table S7.** Multivariable analysis of associated factors with the increased *frequency* of e-cigarettes used during Shelter-in-Place orders: Results from *penalized* logistic regression models adjusted for demographics (N=84)

| **Characteristic** | **Model 1** | **Model 2** | **Model 3** | **Model 4** | **Model 5** | **Model 6** | **Model 7** |
| --- | --- | --- | --- | --- | --- | --- | --- |
| **Sociodemographic characteristics / Main predictor of interest** | AOR (95% CI)^a^,  *p*-value | | | | | | |
| Age | 1.88 (1.20-3.18), p=.01* | 1.92 (1.22-3.25), p=.01* | 1.96 (1.26-3.32), p=.007** | 2.00 (1.28-3.39), p=.006** | 2.26 (1.38-4.13), p=.003** | 1.95 (1.22-3.41), p=.01* | 1.92 (1.25-3.19), p=.007** |
| **Race-ethnicity** |  |  |  |  |  |  |  |
| African American/Black, non-Hispanic | 0.67 (0.12-3.97), p=.66 | 1.05 (0.21-5.88), p=.96 | 1.01 (0.20-5.74), p=.99 | 0.92 (0.17-5.38), p=.93 | 0.82 (0.14-5.24), p=.83 | 0.70 (0.12-4.42), p=.70 | 0.97 (0.20-5.24), p=.97 |
| Hispanic, any race | 0.33 (0.08-1.19), p=.11 | 0.36 (0.10-1.23), p=.12 | 0.52 (0.15-1.7), p=.30 | 0.37 (0.10-1.25), p=.13 | 0.37 (0.10-1.25), p=.13 | 0.47 (0.13-1.64), p=.26 | 0.45 (0.13-1.46), p=.20 |
| Other | 0.83 (0.14-5.84), p=.85 | 1.35 (0.25-9.17), p=.75 | 1.27 (0.23-8.86), p=.80 | 1.06 (0.18-7.67), p=.95 | 0.97 (0.17-6.54), p=.97 | 1.03 (0.18-7.45), p=.98 | 1.22 (0.23-8.07), p=.83 |
| White, non-Hispanic | Reference | Reference | Reference | Reference | Reference | Reference | Reference |
| **Mother’s educational attainment** |  |  |  |  |  |  |  |
| (Some) College degree | 0.95 (0.26-3.34), p=.93 | 0.95 (0.25-3.44), p=.94 | 0.77 (0.20-2.79), p=.70 | 0.64 (0.15-2.44), p=.54 | 0.77 (0.19-2.87), p=.71 | 0.69 (0.15-2.88), p=.62 | 0.96 (0.26-3.44), p=.96 |
| GED^c^/High School or below | 1.59 (0.38-7.02), p=.54 | 1.79 (0.40-8.61), p=.46 | 1.29 (0.29-5.75), p=.74 | 1.04 (0.21-4.98), p=.96 | 2.31 (0.49-11.87), p=.31 | 1.15 (0.24-5.61), p=.86 | 1.50 (0.35-6.64), p=.59 |
| Unknown | 1.23 (0.11-14.77), p=.87 | 0.97 (0.09-9.8), p=.98 | 0.47 (0.05-3.9), p=.52 | 0.61 (0.06-5.04), p=.67 | 0.67 (0.07-6.23), p=.74 | 0.27 (0.02-2.87), p=.31 | 0.59 (0.06-5.01), p=.66 |
| (Some) Graduate or professional degree | Reference | Reference | Reference | Reference | Reference | Reference | Reference |
| Saw e-cigarette social media content | 2.61 (0.74-10.10), p=.16 | N/A | N/A | N/A | N/A | N/A | N/A |
| E-cigarette dependence | N/A | 1.12 (1.00-1.27), p=.06 | N/A | N/A | N/A | N/A | N/A |
| A sibling knows the respondent uses e-cigarettes | N/A | N/A | 2.41 (0.93-6.55), p=.09 | N/A | N/A | N/A | N/A |
| **No of family members who know you use e-cigarettes** |  |  |  |  |  |  |  |
| No one | Reference | Reference | Reference | Reference | Reference | Reference | Reference |
| One person | N/A | N/A | N/A | 2.47 (0.53-13.11), p=.29 | N/A | N/A | N/A |
| Two or more | N/A | N/A | N/A | 5.70 (1.11-34.91), p=.06 | N/A | N/A | N/A |
| E-cigarette use to cope with COVID-19 | N/A | N/A | N/A | N/A | 4.21 (1.39-14.58), p=.02* | N/A | N/A |
| Feeling lonely all or most of the time | N/A | N/A | N/A | N/A | N/A | 5.04 (1.73-17.44), p=.006** | N/A |
| Not able to meet up/hang out with the people | N/A | N/A | N/A | N/A | N/A | N/A | 0.69 (0.26-1.81), p=.47 |

Notes: Sensitivity analysis 3a.

^a^AOR: Adjusted odds ratio; CI: confidence interval

^b^GED: General Education Development Test

^c^N/A: Not applicable

*– Indicates statistically significant results (p≤.05); ** – Bonferroni significance level to account for carrying out seven models (.05/7): p≤.007

# **Table S8.** Multivariable analysis of associated factors with the increased *frequency* of e-cigarettes used during Shelter-in-Place orders: Results from *traditional* logistic regression models adjusted for demographics (N=84)

| **Characteristic** | **Model 1** | **Model 2** | **Model 3** | **Model 4** | **Model 5** | **Model 6** | **Model 7** |
| --- | --- | --- | --- | --- | --- | --- | --- |
| **Sociodemographic characteristics / Main predictor of interest** | AOR (95% CI)^a^,  *p*-value | | | | | | |
| Age | 2.08 (1.23-3.51), p=.006** | 2.13 (1.26-3.60), p=.005** | 2.19 (1.30-3.68), p=.003** | 2.25 (1.33-3.82), p=.003** | 2.61 (1.44-4.73), p=.002** | 2.18 (1.26-3.79), p=.006** | 2.12 (1.29-3.50), p=.003** |
| **Race-ethnicity** |  |  |  |  |  |  |  |
| African American/Black, non-Hispanic | 0.65 (0.10-4.18), p=.65 | 1.11 (0.19-6.56), p=.91 | 1.07 (0.18-6.34), p=.94 | 0.93 (0.14-5.98), p=.94 | 0.83 (0.12-5.87), p=.86 | 0.70 (0.10-4.69), p=.71 | 1.02 (0.18-5.70), p=.98 |
| Hispanic, any race | 0.29 (0.07-1.17), p=.08 | 0.31 (0.08-1.19), p=.09 | 0.49 (0.14-1.73), p=.27 | 0.32 (0.08-1.23), p=.10 | 0.32 (0.09-1.21), p=.09 | 0.43 (0.11-1.67), p=.22 | 0.41 (0.12-1.46), p=.17 |
| Other | 0.90 (0.12-6.58), p=.92 | 1.60 (0.23-11.15), p=.64 | 1.49 (0.21-10.6), p=.69 | 1.18 (0.16-8.96), p=.87 | 1.07 (0.15-7.59), p=.94 | 1.15 (0.15-8.68), p=.89 | 1.41 (0.21-9.44), p=.73 |
| White, non-Hispanic | Reference | Reference | Reference | Reference | Reference | Reference | Reference |
| **Mother’s educational attainment** |  |  |  |  |  |  |  |
| (Some) College degree | 0.91 (0.23-3.56), p=.90 | 0.92 (0.23-3.70), p=.91 | 0.72 (0.18-2.90), p=.64 | 0.57 (0.13-2.58), p=.47 | 0.71 (0.17-2.97), p=.64 | 0.63 (0.13-3.05), p=0.57 | 0.94 (0.24-3.69), p=.93 |
| GED^c^/High School or below | 1.69 (0.36-7.90), p=.51 | 1.97 (0.39-9.99), p=.41 | 1.31 (0.27-6.29), p=.73 | 1.02 (0.19-5.55), p=.98 | 2.67 (0.49-14.48), p=.26 | 1.16 (0.22-6.12), p=.86 | 1.59 (0.34-7.44), p=.56 |
| Unknown | 1.24 (0.09-17.74), p=0.87 | 0.94 (0.08-11.67), p=.96 | 0.40 (0.04-4.20), p=.44 | 0.52 (0.05-5.66), p=.59 | 0.60 (0.05-6.97), p=0.68 | 0.21 (0.01-2.95), p=.25 | 0.52 (0.05-5.51), p=.59 |
| (Some) Graduate or professional degree | Reference | Reference | Reference | Reference | Reference | Reference | Reference |
| Saw e-cigarette social media content | 3.03 (0.76-12.15), p=.12 | N/A | N/A | N/A | N/A | N/A | N/A |
| E-cigarette dependence | N/A | 1.14 (1.01-1.30), p=.04* | N/A | N/A | N/A | N/A | N/A |
| A sibling knows the respondent uses e-cigarettes | N/A | N/A | 2.72 (0.96-7.71), p=.06 | N/A | N/A | N/A | N/A |
| **No of family members who know you use e-cigarettes** |  |  |  |  |  |  |  |
| No one | Reference | Reference | Reference | Reference | Reference | Reference | Reference |
| One person | N/A | N/A | N/A | 2.94 (0.53-16.42), p=.22 | N/A | N/A | N/A |
| Two or more | N/A | N/A | N/A | 7.74 (1.20-49.81), p=.03* | N/A | N/A | N/A |
| E-cigarette use to cope with COVID-19 | N/A | N/A | N/A | N/A | 5.42 (1.53-19.21), p=.009* | N/A | N/A |
| Feeling lonely all or most of the time | N/A | N/A | N/A | N/A | N/A | 6.58 (1.89-22.89), p=.003** | N/A |
| Not able to meet up/hang out with the people | N/A | N/A | N/A | N/A | N/A | N/A | 0.66 (0.24-1.83), p=.42 |

Notes: Sensitivity analysis 3b.

^a^AOR: Adjusted odds ratio; CI: confidence interval

^b^GED: General Education Development Test

^c^N/A: Not applicable

*– Indicates statistically significant results (p≤.05); ** – Bonferroni significance level to account for carrying out seven models (.05/7): p≤.007

# **Table S9.** Multivariable analysis of associated factors with the increased *concentration* of e-cigarettes used during Shelter-in-Place orders: Results from *penalized* logistic regression models adjusted for demographics (N=84)

| **Characteristic** | **Model 1** | **Model 2** | **Model 3** | **Model 4** | **Model 5** | **Model 6** | **Model 7** |
| --- | --- | --- | --- | --- | --- | --- | --- |
| **Sociodemographic characteristics / Main predictor of interest** | AOR (95% CI)^a^,  *p*-value | | | | | | |
| Age | 1.32 (0.90-2.01), p=.19 | 1.34 (0.90-2.05), p=.18 | 1.41 (0.97-2.14), p=.10 | 1.41 (0.96-2.17), p=.11 | 1.47 (1.00-2.28), p=0.08 | 1.34 (0.92-2.05), p=.16 | 1.42 (0.97-2.17), p=.10 |
| **Race-ethnicity** |  |  |  |  |  |  |  |
| African American/Black, non-Hispanic | 1.54 (0.36-6.82), p=.58 | 2.28 (0.53-10.23), p=.29 | 2.18 (0.54-9.22), p=.30 | 1.57 (0.34-7.58), p=.58 | 1.74 (0.39-7.81), p=.48 | 1.92 (0.46-8.42), p=.39 | 2.25 (0.55-9.68), p=.28 |
| Hispanic, any race | 1.27 (0.38-4.31), p=.71 | 1.32 (0.40-4.43), p=.66 | 1.81 (0.57-5.96), p=.34 | 1.31 (0.39-4.51), p=.67 | 1.59 (0.50-5.21), p=.45 | 1.86 (0.57-6.31), p=.33 | 1.65 (0.51-5.55), p=.42 |
| Other | 1.49 (0.31-7.33), p=.63 | 2.16 (0.43-11.46), p=.38 | 2.03 (0.44-9.72), p=.39 | 1.34 (0.28-6.64), p=.73 | 1.81 (0.39-8.76),  p=.47 | 1.98 (0.43-9.7), p=.41 | 1.88 (0.40-9.21), p=.45 |
| White, non-Hispanic | Reference | Reference | Reference | Reference | Reference | Reference | Reference |
| **Mother’s educational attainment** |  |  |  |  |  |  |  |
| (Some) College degree | 0.91 (0.29-2.93), p=.88 | 0.95 (0.29-3.19), p=.94 | 0.76 (0.24-2.48), p=.66 | 0.51 (0.12-1.93), p=.35 | 0.83 (0.25-2.72), p=.76 | 0.82 (0.25-2.71), p=.75 | 1.06 (0.32-3.54), p=.93 |
| GED^c^/High School or below | 1.34 (0.36-5.05), p=.68 | 1.52 (0.39-6.21), p=.56 | 1.09 (0.29-4.08), p=.90 | 0.65 (0.14-2.74), p=.58 | 1.68 (0.43-6.98), p=.48 | 1.14 (0.3-4.39), p=.86 | 1.31 (0.35-5.09), p=.70 |
| Unknown | 1.53 (0.16-14.72), p=.73 | 1.46 (0.16-13.4), p=.75 | 0.63 (0.08-4.46), p=.67 | 0.67 (0.07-6.02), p=.73 | 0.85 (0.1-6.51), p=.88 | 0.53 (0.06-4.08), p=.58 | 0.75 (0.09-5.87), p=.80 |
| (Some) Graduate or professional degree | Reference | Reference | Reference | Reference | Reference | Reference | Reference |
| Saw e-cigarette social media content | 2.75 (0.84-10.23), p=.12 | N/A | N/A | N/A | N/A | N/A | N/A |
| E-cigarette dependence | N/A | 1.18 (1.06-1.33), p=.007** | N/A | N/A | N/A | N/A | N/A |
| A sibling knows the respondent uses e-cigarettes | N/A | N/A | 1.73 (0.72-4.26), p=.25 | N/A | N/A | N/A | N/A |
| **No of family members who know you use e-cigarettes** |  |  |  |  |  |  |  |
| No one | Reference | Reference | Reference | Reference | Reference | Reference | Reference |
| One person | N/A | N/A | N/A | 19.54 (2.12-2620.89), p=.047* | N/A | N/A | N/A |
| Two or more | N/A | N/A | N/A | 25.34 (2.58-3449.29), p=.03* | N/A | N/A | N/A |
| E-cigarette use to cope with COVID-19 | N/A | N/A | N/A | N/A | 3.04 (1.12-9.21), p=.04* | N/A | N/A |
| Feeling lonely all or most of the time | N/A | N/A | N/A | N/A | N/A | 2.86 (1.16-7.28), p=.03* | N/A |
| Not able to meet up/hang out with the people | N/A | N/A | N/A | N/A | N/A | N/A | 0.38 (0.15-0.92), p=.04* |

Notes: Sensitivity analysis 4a.

^a^AOR: Adjusted odds ratio; CI: confidence interval.

^b^GED: General Education Development Test.

^c^N/A: Not applicable.

^d^ – The maximum likelihood estimate may not exist. Results shown are based on the last maximum likelihood iteration. Validity of the model fit is questionable.

*– Indicates statistically significant results (p≤.05); ** – Bonferroni significance level to account for carrying out seven models (.05/7): p≤.007

# **Table S10.** Multivariable analysis of associated factors with the increased *concentration* of e-cigarettes used during Shelter-in-Place orders: Results from *traditional* logistic regression models adjusted for demographics (N=84)

| **Characteristic** | **Model 1** | **Model 2** | **Model 3** | **Model 4** | **Model 5** | **Model 6** | **Model 7** |
| --- | --- | --- | --- | --- | --- | --- | --- |
| **Sociodemographic characteristics / Main predictor of interest** | AOR (95% CI)^a^,  *p*-value | | | | | | |
| Age | 1.38 (0.90-2.11), p=.14 | 1.40 (0.90-2.17), p=.13 | 1.48 (0.98-2.25), p=.06 | 1.49 (0.96-2.30),  p=.07 | 1.56 (1.00-2.42), p=.048* | 1.41 (0.92-2.15), p=.12 | 1.49 (0.97-2.29), p=.07 |
| **Race-ethnicity** |  |  |  |  |  |  |  |
| African American/Black, non-Hispanic | 1.60 (0.34-7.47), p=.55 | 2.47 (0.52-11.74), p=.25 | 2.36 (0.53-10.44), p=.26 | 1.63 (0.31-8.41),  p=.56 | 1.81 (0.37-8.75), p=.46 | 2.04 (0.44-9.42), p=.36 | 2.43 (0.54-10.91), p=.25 |
| Hispanic, any race | 1.29 (0.36-4.57), p=.70 | 1.32 (0.37-4.67), p=.67 | 1.93 (0.57-6.54), p=.29 | 1.35 (0.37-4.87),  p=.65 | 1.65 (0.49-5.57), p=.42 | 1.98 (0.57-6.95), p=.28 | 1.73 (0.50-6.02), p=.39 |
| Other | 1.58 (0.30-8.30), p=.59 | 2.4 (0.42-13.76), p=.32 | 2.20 (0.43-11.17), p=.34 | 1.39 (0.26-7.45),  p=.70 | 1.93 (0.37-10.00), p=.43 | 2.14 (0.41-11.11), p=.37 | 2.03 (0.39-10.63), p=.40 |
| White, non-Hispanic | Reference | Reference | Reference | Reference | Reference | Reference | Reference |
| **Mother’s educational attainment** |  |  |  |  |  |  |  |
| (Some) College degree | 0.91 (0.27-3.08), p=.88 | 0.96 (0.27-3.38), p=.94 | 0.75 (0.22-2.56), p=.64 | 0.45 (0.10-1.99),  p=.29 | 0.81 (0.23-2.83), p=.75 | 0.81 (0.23-2.85), p=.74 | 1.08 (0.31-3.80), p=.91 |
| GED^c^/High School or below | 1.38 (0.35-5.49), p=.65 | 1.64 (0.38-7.01), p=.51 | 1.10 (0.28-4.37), p=.89 | 0.60 (0.12-2.87),  p=.52 | 1.84 (0.42-7.98), p=.42 | 1.16 (0.28-4.71), p=.84 | 1.38 (0.34-5.62), p=.66 |
| Unknown | 1.61 (0.14-18.05), p=.70 | 1.52 (0.14-16.30), p=.73 | 0.57 (0.07-4.94), p=.61 | 0.60 (0.05-6.85),  p=.68 | 0.81 (0.09-7.47), p=.85 | 0.48 (0.05-4.47), p=.52 | 0.70 (0.07-6.63), p=.76 |
| (Some) Graduate or professional degree | Reference | Reference | Reference | Reference | Reference | Reference | Reference |
| Saw e-cigarette social media content | 3.17 (0.85-11.9), p=.09 | N/A | N/A | N/A | N/A | N/A | N/A |
| E-cigarette dependence | N/A | 1.21 (1.06-1.36), p=.003** | N/A | N/A | N/A | N/A | N/A |
| A sibling knows the respondent uses e-cigarettes | N/A | N/A | 1.84 (0.72-4.70), p=.20 | N/A | N/A | N/A | N/A |
| **No of family members who know you use e-cigarettes** |  |  |  |  |  |  |  |
| No one | Reference | Reference | Reference | Reference | Reference | Reference | Reference |
| One person | N/A | N/A | N/A | 613604.57 (0-8.1733249090883E202), p=.95^d^ | N/A | N/A | N/A |
| Two or more | N/A | N/A | N/A | 821259.33 (0-1.0942622393994E203), p=.95^d^ | N/A | N/A | N/A |
| E-cigarette use to cope with COVID-19 | N/A | N/A | N/A | N/A | 3.59 (1.17-10.99), p=.03* | N/A | N/A |
| Feeling lonely all or most of the time | N/A | N/A | N/A | N/A | N/A | 3.23 (1.22-8.51), p=.02* | N/A |
| Not able to meet up/hang out with the people | N/A | N/A | N/A | N/A | N/A | N/A | 0.34 (0.13-0.88), p=.03* |

Notes: Sensitivity analysis 4b.

^a^AOR: Adjusted odds ratio; CI: confidence interval.

^b^GED: General Education Development Test.

^c^N/A: Not applicable.

^d^ – The maximum likelihood estimate may not exist. Results shown are based on the last maximum likelihood iteration. Validity of the model fit is questionable.

*– Indicates statistically significant results (p≤.05); ** – Bonferroni significance level to account for carrying out seven models (.05/7): p≤.007
